# Gender differences in palliative care needs among Swedish cancer patients prior to specialist palliative care referral

**DOI:** 10.2340/1651-226X.2025.43308

**Published:** 2025-05-14

**Authors:** Karin Boo Hammas, Juliet Jacobsen, Rebecca Selberg, Sanjoy Mahajan, Jenny Klintman

**Affiliations:** a Department of Clinical Sciences Lund, Medical Oncology, Lund University, Lund, Sweden; b Department of Specialised Palliative Care, Advanced Home Health Care, Kristianstad, Sweden; c Harvard Medical School and Massachusetts General Hospital, Boston, MA, USA; d Department of Sociology, Lund University, Lund, Sweden; e Centre for Mathematical Sciences, Lund University, Lund, Sweden; f The Institute for Palliative Care, Lund, Sweden

**Keywords:** Palliative care, gender equity, healthcare disparities, health communication

## Abstract

Background and purpose: Few studies, in Sweden or internationally, have examined gender differences regarding the use of palliative care. This study investigates gender differences in palliative care needs prior to referral in a regional cohort of Swedish cancer patients.

Patient/material and methods: Adult cancer patients who died throughout 1 year and were referred to a specialized palliative care service in southern Sweden during their last 3 years of life (n = 192) were included. Information on gender, age, diagnosis, performance status, admissions to hospital, and serious illness conversations was collected through chart review.

Results: Ninety-nine (52%) women and 93 (48%) men were included. Survival from diagnosis until death was comparable (p = 0.27) for women (341.0 days, IQR 77.0–902.0) and men (463.0 days, IQR 141.0–1035.0), as was survival from palliative care referral (p = 0.06) (women 48.0 days, IQR 19.0‑107.5; men 36.0 days, IQR 17.0‑85.0). Performance status at the time of referral was also comparable (p = 0.59). Gender differences were observed in healthcare utilization with fewer hospitalizations and emergency department visits for women in the 6 months prior to referral (p = 0.03) and significantly more men among those with the highest healthcare utilization (≥4 hospitalizations and emergency department visits) (p = 0.005). During the month before referral, women were more likely to have a serious illness conversation (p = 0.01).

Interpretation: Compared to women, men have more hospitalizations and fewer serious illness conversations prior to referral to specialized palliative care, suggesting greater unmet palliative care needs.

## Introduction

Previous studies on gender differences in end-of-life care (mostly conducted in the UK and USA [1]) suggest that men have greater unmet palliative care needs. Men are less likely to communicate about serious illness. For example, men diagnosed with advanced cancer are less likely to state that their tumor is incurable or to have a conversation about prognosis [2] or mortality [3]. Men more frequently make treatment decisions at the end of life that are associated with more aggressive, generally nonbeneficial care [4]. Men in the last stages of life are more likely to receive intensive care unit treatment, cardiopulmonary resuscitation, mechanical ventilation, hemodialysis, and surgical procedures [5–7]. Similarly, men in the last days of life are more likely to receive chemotherapy and less likely to request palliative care [8] or to receive hospice care [7].

In Sweden, few studies focus on gender differences in palliative care, and the findings are somewhat contradictory. On one hand, men and women who wish to die at home do so to the same extent [9]. For patients with ALS, there is no gender difference in access to palliative care [10], and men and women with brain tumors have comparable access to specialized palliative care (SPC) in the last month of life although men are more likely to be hospitalized [11]. However, in hematologic malignancies, women were more likely to receive SPC during the last 3 months of life [12]. Contrary to the American findings, Swedish women tend to receive more aggressive care than men. Swedish women are more likely than men to receive palliative chemotherapy [13], and women with esophageal cancer spend a higher proportion of their time hospitalized than men in the first year after diagnosis [14].

These studies focused on end-of-life care. As the value of palliative care is increasingly recognized and evidence supports earlier integration of palliative care [15], there is a need to investigate barriers to care access (including gender) in healthcare settings outside of SPC. Thus, we aimed to investigate gender differences in primary palliative care needs of cancer patients, focusing on timing of SPC-referral, healthcare utilization, and communication about serious illness prior to referral.

## Patients/material and methods

This is a retrospective observational study. Region Skane has one regional provider of comprehensive SPC with eight local sites. This study was conducted in one local site with a catchment area population of approximately 110,000 inhabitants. Hospital care is provided by a 24-h-service regional hospital with approximately 250 beds and 2,500 staff. The study included all adults with a diagnosis of incurable cancer who died between 1 September 2022 and 31 August 2023 and who had been referred to the local SPC unit in the last 3 years of life (*n* = 192). Using chart review, we collected the date of the last systemic anticancer treatment before death and baseline information at referral: gender, age, living situation, date of primary cancer diagnosis, and performance status (ECOG). We also collected Supportive and Palliative Care Indicators Tool (SPICT) indicators of palliative care needs (general indicators of poor or deteriorating health and clinical indicators of serious illnesses or health conditions) [16], unplanned hospitalizations, and emergency department (ED) visits during the 6 months prior to referral to SPC (or from date of diagnosis if cancer diagnosis was <6 months before referral). Finally, because serious illness communication (SIC) is important for advance care planning and goals-of-care discussions [17], we screened for four key components of an SIC (information about disease progression/prognosis, patient priorities, treatment options/plan in case of deterioration/withholding of life-sustaining treatment, and options for symptom relief) during the month before referral to SPC. Referring clinicians were from a wide range of clinics including oncology, gynecology, lung, surgery, and urology. Gender of the referring clinician was not collected. Chart review was done by the first author, an experienced primary and palliative care physician (KBH).

### Data analysis and statistics

Data were collected and stored in REDCap. Descriptive information on background characteristics of the study group is presented as frequencies, percentages, median, and interquartile range (IQR). Comparisons of medians between women and men were performed using the Mann‑Whitney test for continuous parameters and Fisher’s exact test and the chi-squared test with Yates’ continuity correction for proportions. A *p* < 0.05 was considered statistically significant. Statistical analyses were made in Microsoft Excel (version 2402 for Windows, Microsoft Corporation, Redmond, WA, USA), with the programming language R (version 4.3.0, R Core Team (2023) R Foundation for Statistical Computing, Vienna, Austria) and with GraphPad Prism (version 9.1.2, GraphPad Software Inc., La Jolla, California, USA).

## Results

**Figure 1:**
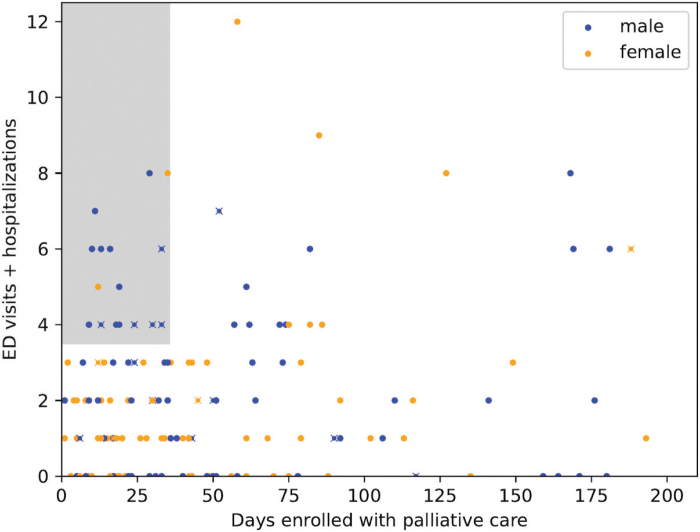
Healthcare utilization in a subset of patients with ≤200 survival days from SPC referral (n = 153: n women 76, n men 77). Significantly more men (blue) than women (yellow) had ≥4 ED visits/hospitalizations (n = 24, 31.2% of 77 men and n = 9, 11.8% of 76 women, p = 0.005). Men (blue) were the most of patients (n men = 14/16, 87.5%) with the highest palliative care needs defined as highest utilization of unplanned hospital care (≥4 ED visits/hospitalizations), latest palliative care referral (≤35 days before death), and no documented SIC (n = 5/14 of men, 35.7%) (grey box, those with an x).

Ninety-nine women (52%) and 93 men (48%) were included (Table 1). All patients fulfilled ≥1 general SPICT indicator and the clinical indicator related to cancer (i.e. progressive disease or no curative treatment intent). The most common cancer forms were upper gastrointestinal (*n* = 46, 24%), colorectal (*n* = 29, 15%), and lung (*n* = 21, 11%) (Table 1). The median age at referral was 75.9 years. Most women (*n* = 89, 90%) and men (*n* = 88, 95%) were living at home; 49/89 women (49%) and 33/88 men (35%) lived alone (*p* = 0.07). The median number of days from the last systemic anticancer treatment until death was similar in women (64.5 days; IQR 37.8‑178.0) and men (66.5 days; IQR 38.5‑127.0, *p* = 0.67). Median days were comparable between women and men from referral to SPC until death (women 48.0 days (IQR 19.0‑107.5) and men 36.0 days (IQR 17.0‑85.0, *p* = 0.06) and from primary cancer diagnosis until death (women 341 days; IQR 77.0‑902.0) and men (463.0 days; IQR 141.0‑1035.0, *p* = 0.27). There was no gender difference in performance status (WHO/ECOG) at referral (*p* = 0.59). Six months prior to referral to SPC, the median number of ED visits/hospitalizations was significantly fewer for women (1.0; IQR 0.0‑2.0) than men (2.0; IQR 0.0‑3.0, *p* = 0.03). In a subset analysis of healthcare utilization in patients with ≤200 survival days from SPC referral (*n* = 153: n women 76 and *n* men 77), significantly more men (*n* = 24, 31.2% of 77 men and *n* = 9, 11.8% of 76 women, *p =* 0.005) had ≥4 ED visits/hospitalizations (Figure 1).

**Table 1:** Background characteristics of the study cohort.

Variables investigated		Total	Women	Men	*p* *
Included patients (*n*, %)		192	99 (52)	93 (48)	na
Median age at first referral (IQR) (*n* = 192)		75.9 (68.5–82.5)	76.5 (68.4–84.2)	74.8 (69.4–81.6)	0.55
Residing in own home (*n*, %) (*n* = 192)		177 (92)	89 (90)	88 (95)	0.29
Nursing home resident (*n*, %) (*n* = 192)		15 (8)	10 (10)	5 (5)	0.29
Living alone (n, %) (*n* = 192)		82 (43)	49 (49)	33 (35)	0.07
Median survival from cancer diagnosis (days, IQR) (*n* = 192)		395.0 (103–1020)	341.0 (77.0–902.0)	463.0 (141.0–1035.0)	0.27
Median survival from last systemic anticancer treatment (days, IQR) (*n* = 122)^a^		65.5 (37.8–161.5)	64.5 (37.8–178.0, *n* = 58)	66.5 (38.5–127.0, *n* = 64)	0.67
Cancer diagnosis (*n*, %) (*n* = 192)	Upper GI, bile, pancreatic	46 (24.0)	26 (56.5)	20 (43.5)	na
	Colorectal	29 (15.1)	9 (31.0)	20 (69.0)	na
	Lung	21 (10.9)	13 (61.9)	8 (30.1)	na
	Prostate	17 (8.9)	na	17	na
	Gynecological	13 (6.8)	13	na	na
	Hematological	12 (6.3)	8 (66.7)	4 (33.3)	na
	Breast	11 (5.7)	11	na	na
	CNS	10 (5.2)	4 (40.0)	6 (60.0)	na
	Unknown primary	9 (4.7)	6 (66.7)	3 (33.3)	na
	Urological	6 (3.1)	0	6 (100)	na
	Ear, nose, throat	6 (3.1)	2 (33.3)	4 (66.7)	na
	Hepatocellular	5 (2.6)	3 (60.0)	2 (40.0)	na
	Melanoma	5 (2.6)	2 (40.0)	3 (60.0)	na
	Sarcoma	2 (1.0)	2 (100)	0	na
ECOG at referral (median, IQR) (*n* = 185)^b^		3.0 (3.0–3.0)	3.0 (3.0–3.0, *n* = 96)	3.0 (2.0–3.0, *n* = 89)	0.59
Hospital admissions and ED visits <6 months prior to the first PC referral (median, IQR) (*n* = 192)		1.0 (0.0–3.0)	1.0 (0.0–2.0)	2.0 (0.0–3.0)	0.03
Median survival from SPC referral (days, IQR) (*n* = 182)^c^		42.0 (17.0–95.0)	48.0 (19.0–107.5, *n* = 95)	36.0 (17.0–85.0, *n* = 87)	0.06

* Comparisons were performed using the Mann‑Whitney test for continuous parameters and Fisher’s exact test for proportions.

a Includes patients who received systemic anticancer treatment with palliative intent.

b ECOG performance status was not assessable for seven patients

c Includes patients referred for assessment of full enrolment in specialized palliative care. Patients referred for limited care provision (e.g. erythrocyte transfusion, antibiotic treatment, etc.) were not included in this analysis.

GI: gastrointestinal; CNS: central nervous system; ECOG: Eastern Cooperative Oncology Group; ED: emergency department; SPC: specialized palliative care; IQR: interquartile range.

Women were more likely than men to have documentation of at least one of four components of an SIC prior to SPC referral (*p* = 0.01). More women than men had a documented conversation about disease progression/prognosis (*p* = 0.02) and priorities (*p* = 0.049) (Table 2). When adjusted for living alone, the gender difference was still significant regarding total frequency of SIC (*p* = 0.01). Finally, we analyzed SIC in a subset of patients with the highest healthcare utilization (≥4 ED visits/hospitalizations) and latest SPC referral (≤35 days before death) (*n* = 16, grey box Figure 1) in which men were the majority (*n* = 14, 87.5%) and among which 35.7% (*n* = 5/14) had no documented SIC.

**Table 2:** Frequency of conversation about serious illness during the month prior to a palliative care referral.

SIC components	Total	Women	Men	*p*-value*
Information regarding prognosis/disease progression (*n*, %) (*n* = 192)	140 (72.9)	80 (80.8)	60 (64.5)	0.02
Patient priorities (*n*, %) (*n* = 192)	66 (34.4)	41 (41.4)	25 (26.9)	0.049
Treatment in case of deterioration/withholding of life-sustaining treatment (*n*, %) (*n* = 192)	79 (41.1)	47 (47.5)	32 (34.4)	0.09
Symptom relief (*n*, %) (*n* = 192)	95 (49.5)	56 (56.6)	39 (41.9)	0.06
Conversation about any of the four themes (*n*, %) (*n* = 192)	163 (81.9)	91 (91.9)	72 (77.4)	0.01

* Comparisons were performed using the chi-squared test with Yates’ continuity correction.

## Discussion and conclusion

We present an investigation of gender differences in palliative care needs prior to SPC referral in a regional Swedish cancer cohort, and we find men having more unmet palliative care needs than women. Compared to women, men had more ED visits and hospital admissions and fewer documented SICs prior to SPC referral.

We did not find any clear structural factors that explain these gender differences. The groups were similar in age, living accommodations, time of last systemic anticancer treatment, and performance status. Because survival from diagnosis was similar for men and women, we could not explain the difference as due to more aggressive disease progression among men. A potential structural difference identified was a trend toward men cohabitating more frequently than women (*p* = 0.07). This difference accords with an earlier study showing that men rely more than women on social support from their partners, while it is important for women to communicate with and receive support from healthcare personnel [14]. Living with a partner could potentially influence healthcare utilization and communication needs. However, after adjusting for living with a partner or not, men still had significantly fewer SICs and a trend toward more healthcare utilization.

The gender distribution in diagnoses could have influenced our results. However, apart from for lung and urothelial cancers, the gender distribution was comparable in the included cancer types and in the number of patients with gendered cancers (gynecological and breast versus prostate) (Table 1). Furthermore, all the included patients fulfilled at least one SPICT criterion for palliative care needs [16]. Even so, only 27% of men (versus 41% of women) had a documented SIC about priorities, goals of care, etc. prior to referral to SPC, which indicates an unmet palliative care need.

Interestingly, our results contradict some Swedish studies that show no gender difference in use of palliative care [9–11]. However, in hematologic malignancies, women were more likely to be enrolled in SPC at the end of life [12], and international studies have shown higher healthcare utilization and less access to palliative care among men [2, 3, 5–8]. Differences could be explained by study design or epidemiologic reasons. Several of the Swedish studies included cohorts with specific diagnosis and uneven gender distribution, whereas our study includes an unselected, consecutively collected, cohort of cancer patients. Furthermore, the healthcare setting may influence the results. Most of the previous Swedish studies were conducted in the capital region of Stockholm, whereas our cohort resides in a more rural area, albeit still rather densely populated, a difference that might affect access to care and referral patterns. SPC is organized differently across healthcare regions in Sweden, which makes nationwide conclusions difficult to draw based on results from regional studies.

An important finding is that men have fewer documented SICs compared to women. This lower conversation rate is both an outcome and a potential mediator of increased healthcare utilization for men. One possible explanation for the lower conversation rate is poor and/or biased communication skills among clinicians. Studies have found that clinicians lack adequate communication skills training [17] and therefore the confidence and ability to engage in SICs. This lacuna could result in a gender-biased communication practice, in which clinicians more easily approach women for conversations [17]. In society in general, women are thought of as better communicators, while men are thought of as less inclined to express and share feelings – sometimes described as women being ‘expressive’ and men ‘instrumental’ in their communication styles [18]. Such perceived differences could be emphasized by physicians who feel insecure in their communication skills to the detriment of male patients. Alternatively, gendered communication practices may reflect patients’ actual communication preferences; one previous study indicated that female patients tend to be more vocal about their needs and preferences than male patients [19]; perhaps men, more than women, avoid conversations about their illness. Gendered communication differences may not only reflect unmet palliative care needs but may also reflect patients’ wishes [20].

In the clinical setting, there is a fine line between respecting patients’ information and communication preferences and enabling informed medical decision making, which necessitates some prognostic awareness of what lies ahead and the potential benefits (and risks) of treatments and other care options, such as palliative care. Our results indicate that by the time men are enrolled in SPC, they have had few SICs and therefore may need more end-of-life preparation and support. More research on the gender gap in communication is needed [21, 22].

A strength of this study is that it includes all cancer patients referred to a single SPC practice within a geographic region. This approach limits selection bias and ensures that the study represents the needs of a population with a range of different cancer types. A limitation is the small cohort that could be disproportionally impacted by local traditions and referral patterns. All patients in this study were referred to SPC, and the results might have differed if we also had included patients never referred at all. Our next step is therefore to expand the analysis with region-wide registry data.

In conclusion, although care equity is one of the main principles in Swedish healthcare, our study suggests a gendered difference in palliative care needs. Prior to referral to SPC, men have more ED visits and hospitalizations and fewer opportunities for SICs than women. These results are an argument for more research and a greater awareness of potential structural biases in the provision of palliative care.

## Data Availability

The data are available upon request from the corresponding author.
